# Anterior Radial Head Dislocation Associated with a Bifocal Fracture of the Ulna: A Bado Type ID Monteggia Fracture–Dislocation?

**DOI:** 10.3390/life15040637

**Published:** 2025-04-11

**Authors:** Flaviu Moldovan

**Affiliations:** Orthopedics—Traumatology Department, Faculty of Medicine, George Emil Palade University of Medicine, Pharmacy, Science, and Technology of Targu Mures, 540142 Targu Mures, Romania; flaviu.moldovan@umfst.ro; Tel.: +40-754-671-886

**Keywords:** Monteggia fracture–dislocation, anterior radial head dislocation, elbow trauma, Bado type I

## Abstract

Monteggia fractures represent complex injuries requiring careful assessment and surgical intervention. This case report presents a rare variation of a Bado type I Monteggia fracture–dislocation that resembles features from the Jupiter subclassification type IID. A 39-year-old male sustained a high-energy injury while riding an all-terrain vehicle, resulting in a proximal segmental ulnar shaft fracture with anterior radial head dislocation. Open reduction and internal fixation (ORIF) of the ulna using a pre-contoured proximal ulna low-contact dynamic compression plate (LC-DCP) successfully restored alignment, leading to spontaneous reduction of the radial head. The postoperative course was uneventful, with satisfactory healing and functional recovery. This case underscores the importance of meticulous ulnar reconstruction in Monteggia fracture–dislocations and contributes to the limited literature on anterior radial head dislocation patterns.

## 1. Introduction

Monteggia fractures are complex injuries involving a fracture of the ulna associated with a radial head dislocation, accounting for approximately 5% of all traumatic pathologies around the elbow [1]. These injuries were first described by Giovanni Battista Monteggia in 1814 and are classified into different types based on the direction of the radial head dislocation and the pattern of the proximal ulnar fracture using the Bado classification as it is the most widely used system [2]. Four primary types are recognized, of which type II with an apex posterior ulnar fracture and a posterior dislocation of the radial head are the most frequently met in the adult population. As a result, in 1991, Jupiter [3] further subclassified type II Monteggia fractures into various subtypes, including type IID, which involves the olecranon and extends to the distal half of the ulna. Bado type I injuries where the radial head is dislocated anteriorly are rare in adults accounting for only 15% of cases with few cases being presented in the scientific literature [4]. Different subtypes are still questionable as no additional classification systems are proposed. Bado type III (anterolateral dislocation of the radial head with lateral angulation of the fractured ulna in the metaphyseal region) and IV (ulna fracture of the middle or proximal third, with an anterior dislocation of the radial head that is associated with an upper radial third fracture distal to the bicipital tuberosity) are extremely rare. Some consider the last type a variant of the type I injuries, which reinforces the idea that potential subtypes are underdiagnosed [5]. Rare patterns still occur in scientific literature highlighting the limited knowledge of these injuries. For example, Mohamed et al. [6] presented a case where an unusual Monteggia type II variant was associated with type I capitellum fracture and LUCL (lateral ulnar collateral ligament) avulsion due to an additional varus force. Another interesting case presented showed a type IIA injury associated with an ipsilateral distal radius fracture classified as Frykman type V. Most adult unusual patterns presented in scientific literature fall into the type II category, with few cases that assess type I injuries where the radial head is dislocated anteriorly [7]. Modi et al. [8] presented a hybrid fracture pattern where an apex anterior diaphyseal fracture of the radius and ulna were associated with a posterior elbow dislocation, thus exhibiting features of both type I and type II Monteggia equivalent lesion. Even a Bado type V pattern was suggested where there is a fracture of the olecranon process and the radial head dislocation is antero-medial, thus supporting the idea of possible subclassifications in anteriorly dislocated patterns [9].

This case study presents an unusual bifocal Bado type I Monteggia fracture–dislocation that extends from the olecranon to the middle third of the diaphysis, making it a rare variation of the known classification systems.

## 2. Case Presentation

### 2.1. General Presentation

A 39-year-old male was presented to the outpatient room with a painful, swollen right elbow after an all-terrain vehicle (ATV) accident. The patient reported a high-energy fall, landing on his outstretched arm with the elbow in a slightly flexed position. He was right-arm dominant without known comorbidities. Clinical examination revealed significant swelling, tenderness, obvious deformity to the elbow and forearm, and restricted range of motion, with an inability to actively supinate or flex the forearm.

**Figure 1:**
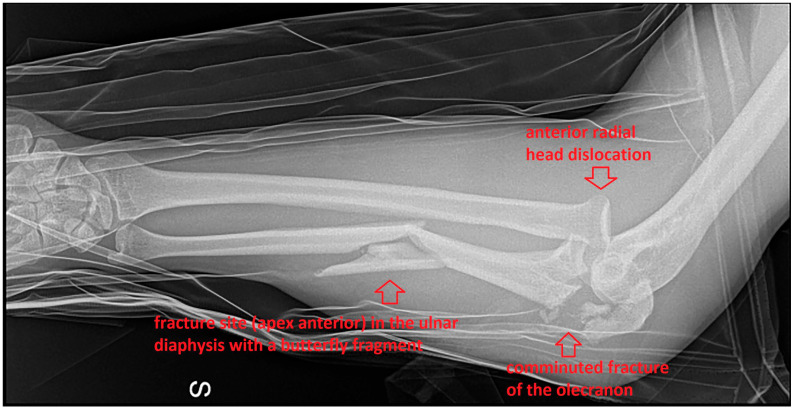
Initial radiograph of the Monteggia lesion.

Initial radiographs of the elbow and forearm revealed a Monteggia lesion with a proximal to middle third segmental fracture of the ulnar shaft contained by a comminuted fracture of the olecranon and distally by a fracture site in the diaphysis with a butterfly fragment, with an atypical anteriorly dislocated radial head (Figure 1).

**Figure 2:**
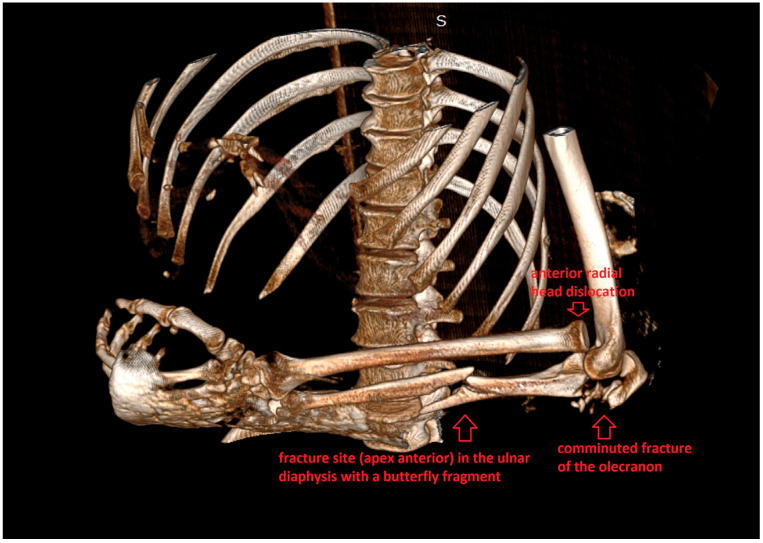
Preoperative 3D CT reconstruction of the Monteggia lesion.

Immobilization in a plaster cast was performed and following thorough preoperative planning with CT imaging (Figure 2), the patient was scheduled for surgical intervention.

### 2.2. Surgical Management

**Figure 3:**
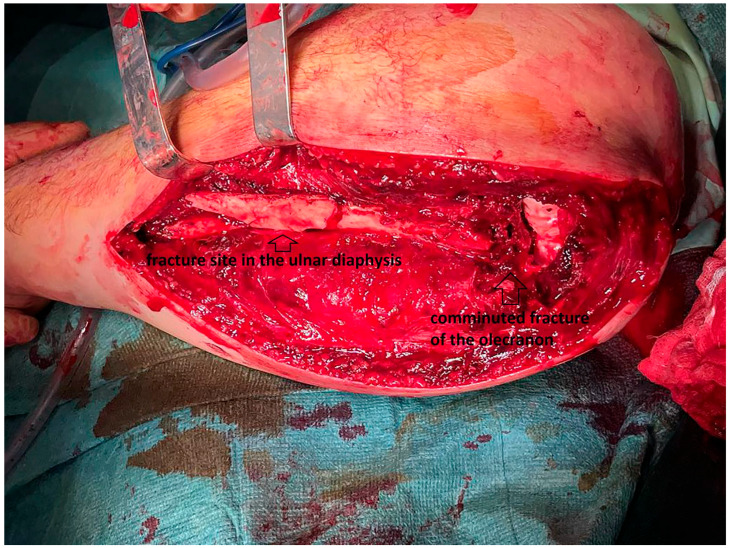
Posterolateral approach to the olecranon and ulnar diaphysis.

The procedure was performed under general anesthesia using a posterolateral approach to the olecranon between the ancouneus and extensor carpi ulnaris, extending the interval distally to the ulnar diaphysis between the extensor and flexor carpi ulnaris (Figure 3).

**Figure 4:**
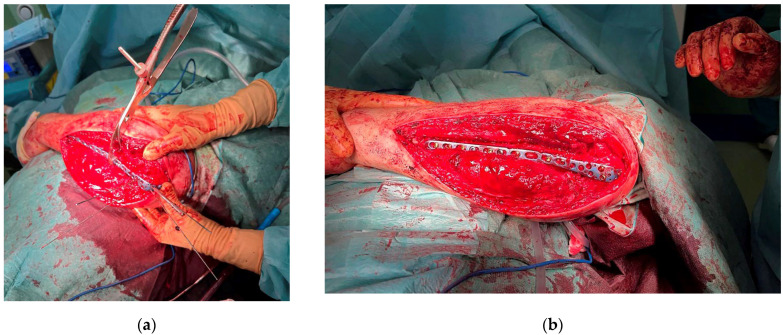
Osteosynthesis construct: (**a**) with temporary K-wires; (**b**) final construct.

Open reduction and internal fixation (ORIF) of the ulna were performed using a pre-contoured proximal ulna low-contact dynamic compression plate (LC-DCP). Due to the significant comminution, temporary K-wires were utilized to aid fracture reduction under plate positioning before definitive fixation with screws was performed (Figure 4). Additionally, Ti-Cron (#2) cerclage sutures were applied to reinforce the construct at the distal fracture site where the butterfly fragment was situated.

**Figure 5:**
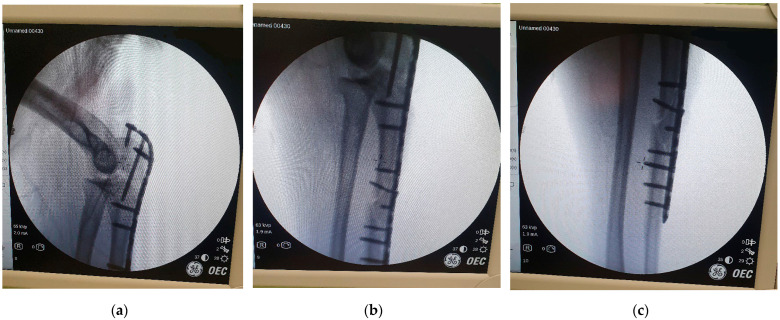
Fluoroscopy assessment of the osteosynthesis construct: (**a**) proximal part; (**b**) middle part; (**c**) distal part.

Intraoperative fluoroscopy confirmed proper reduction and alignment of the ulna. Notably, the reduction of the ulnar fracture led to spontaneous realignment of the radial head without requiring a separate Speed and Boyd approach. The joint was stable upon completion of the osteosynthesis construct, and the radial head maintained its position throughout passive range-of-motion testing (Figure 5).

Postoperatively, the patient’s arm was immobilized in a supinated position with a plaster cast for 2–3 weeks to allow for soft tissue healing and initial fracture consolidation. Standard antibioprophylaxis (with Cefuroxime 1.5 g for three days) was also given.

### 2.3. Postoperative Course and Rehabilitation

**Figure 6:**
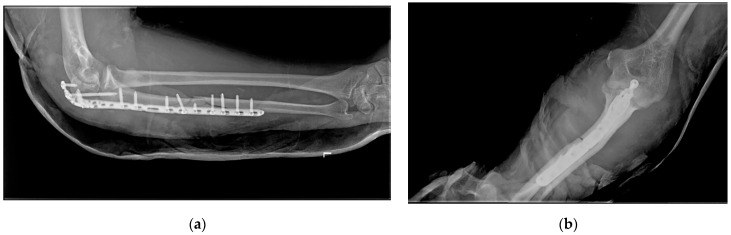
Follow-up radiograph at two weeks: (**a**) lateral view with forearm in neutral position; (**b**) anterio-posterior view with forearm in neutral position.

**Figure 7:**
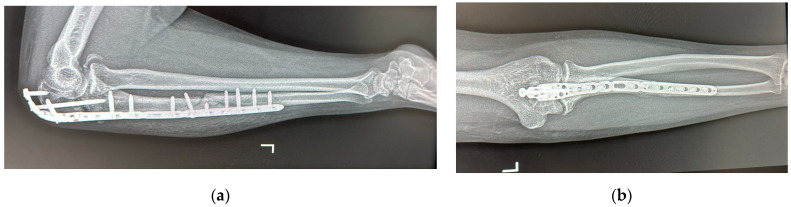
Follow-up radiograph at six weeks: (**a**) lateral view with elbow flexed at 90°; (**b**) anterio-posterior view.

The early postoperative period was uneventful, with no signs of infection or wound healing complications. The patient was advised on a gradual rehabilitation program with physiotherapy aimed at regaining elbow motion and preventing stiffness after the cast that was used for initial soft tissue healing and skin sutures were removed at two weeks. Follow-up radiographs at two (Figure 6) and six weeks (Figure 7) postoperatively demonstrated satisfactory alignment and healing progress. The Mayo Elbow Performance Score at this stage was 58 with a motion arc around 90°. The patient exhibited progressive improvement in elbow flexion and extension, with near-complete restoration of forearm rotation by the three-month follow-up, which was indicated by a Mayo Elbow Performance Score of 88 with a motion arc of over 100°.

Given the complex nature of the injury, continued physiotherapy and serial clinical and radiographic evaluations remain essential to ensure optimal recovery. The patient was informed about the potential long-term complications, including post-traumatic arthritis and restricted range of motion, though no immediate concerns were observed at the latest follow-up.

## 3. Discussion

Monteggia fractures pose a surgical challenge due to their complex anatomy and potential for long-term disability if not adequately managed. The primary goals of surgical treatment include ulnar reconstruction of perfect length, restoration of radial head alignment, and preservation of elbow joint function [10]. In 2022 Tille at al. [11] suggested that in Monteggia-like lesions the location of the ulnar fractures is correlated with the postoperative outcome as intra-articular fractures that involve the coronoid process have a worse prognosis. Another study by Liu et al. [12] suggested that ulnar injuries are of a comminuted pattern with proximal radio-ulnar dislocations more likely to be present if the fracture site is situated in metaphysis or diaphysis. Nonetheless, occult Monteggia fractures have been reported in older individuals where a reduced radio-capitellar joint was seen on initial radiographs [13].

Due to the extension distally into the middle thirds of the ulnar diaphysis, this injury represents a new variant of Bado type I Monteggia fracture. This case was unique in that the expected posterior radial head dislocation was absent, and instead, the patient exhibited an anteriorly dislocated radial head with a segmental fracture of the ulna that started from the olecranon process until the middle third of the shaft, which has not been commonly described in the literature. This case resembles the features of a type IID Jupiter fracture pattern, thus indicating the need to further develop subclassification systems in type I injuries. Such a system exists now just for type II injuries as poorer outcomes were noted by Jupiter in this category due to the associated injuries and greater soft tissue compromise [3].

This unique presentation necessitated careful intraoperative assessment to ensure that the radial head remained stable without additional soft tissue surgical procedures such as annular ligament reconstruction, which was fortunately the case. In rare situations, structures such as the biceps tendon or the brachialis tendon can interfere with reduction of the radial head, but this was seen only in association with an LUCL tear. The posterior interosseus nerve (PIN) was also reported in one case to be trapped in the radiocapitellar joint preventing reduction, with the patient developing a minor weakness while performing resisted extension [14]. The biomechanics behind radial head reduction after the ulna restores its length can be explained by several key anatomical factors: the annular ligament is tensioned, and as it encircles the radial head, it acts like a sling; the proximal radioulnar joint is restored as the radial head re-engages with the radial notch of the ulna; and the biceps and supinator muscles exert forces on the radial tuberosity and radial shaft, further contributing to this alignment.

Hayami et al. [15] used a cadaveric Bado type I Monteggia fracture model to demonstrate that the radial head instability correlated with the degree of ulnar fracture angular deformity and the extent of soft tissue injury. As a result, significant anterior radial head instability was observed in supination after annular ligament sectioning in limbs with moderate ulnar deformity (>5° apex anterior angulation), attributed to interosseous membrane (IOM) loosening. As a clinical implication for anterior irreducible dislocations, Abdelgawad et al. [16] proposed a closed indirect reduction method. This involved creating a flexion moment at the ulnar fracture site using a prebent 20° mini-fragment plate.

New approaches are proposed to treat both injuries in Monteggia patterns to limit soft tissue injuries. Such examples include the modified Boyd approach, which is characterized by using anchors to repair the lateral ligament complex and the annular ligament remains partially intact, or the Wrightington approach, which is characterized by anconeus muscle dissection from and osteotomy of the supinator tuberosity that present excellent exposure of the proximal ulna, the radial head, and the capitellum [17].

The mechanism of injury, although typical for a type II Bado injury, as described by the patient may have involved also hyper-pronated force contributing to an anterior dislocation of the radial head and apex anterior diaphysis fracture of the ulnar [4]. This shift in frequency from type I to type II injuries as the age of the patient increases can be explained as a reduction in bone density that leads to failure earlier than the ligaments in the elbow joint [18]. The outcomes are contradictory in the scientific literature between these two types. Both were seen to be associated with different types of injuries which made the prognosis unforeseeable: type I was associated more with neurological injuries, soft tissue injuries, and ipsilateral fractures, resulting in floating elbows, while type II injuries were linked with fractures of the radial head and coronoid fractures [19,20]. These associated injuries need to be considered when planning the surgical interventions as postoperative complications may occur later. Khadka et al. [21] presented a case report where a proximal radioulnar synostosis following Monteggia fracture–dislocation occurred 9 months after the initial intervention. They highlighted that an excessive trauma-to-surgery interval, significant soft tissue injury, and high-energy kinetic fractures like Bado type I need special considerations. Therefore, Giannacola et al. [22] proposed in 2013 a comprehensive classification system, “the proximal ulnar and radial fracture–dislocation comprehensive classification system (PURCCS)”, with the aim to enhance the surgical management of these difficult injuries.

Future research directions should concentrate on identifying new patterns of injuries, create new subclassification systems, and propose therapeutic algorithms which are integrated into specific workflow protocols. Special considerations should be taken also in correlating these new patterns with associated injuries that may interfere with the final treatment outcomes.

## 4. Conclusions

This case highlights a rare variation of a Bado type I Monteggia fracture–dislocation, with an anterior radial head dislocation and a bifocal fracture of the ulna that has extended distally intro the middle third of the diaphysis. The injury pattern observed suggests that existing classification systems may not fully encompass all variations of Monteggia lesions, warranting further refinement and potential subclassifications especially within Bado type I injuries.

From a clinical perspective, accurate diagnosis requires thorough imaging assessment, including 3D CT reconstruction as early recognition of atypical Monteggia patterns is crucial, as misclassification may result in suboptimal treatment strategies. Long-term follow-up remains essential to monitor complications such as post-traumatic arthritis, restricted motion, and radioulnar synostosis.

By expanding the classification framework and optimizing surgical strategies, clinicians can improve diagnostic accuracy and therapeutic decision-making for complex Monteggia fracture–dislocations, ultimately enhancing patient outcomes.

## Data Availability

The data used in this study can be requested from the corresponding author.
